# Quantitative evaluation of aerosol generation from upper airway suctioning assessed during tracheal intubation and extubation sequences in anaesthetized patients

**DOI:** 10.1016/j.jhin.2022.02.021

**Published:** 2022-06

**Authors:** A.J. Shrimpton, J.M. Brown, T.M. Cook, C.M. Penfold, J.P. Reid, A.E. Pickering

**Affiliations:** a Anaesthesia, Pain and Critical Care, School of Physiology, Pharmacology and Neuroscience, University of Bristol, Bristol, UK; b Department of Anaesthesia and Intensive Care Medicine, North Bristol NHS Trust, Bristol, UK; c Department of Anaesthesia and Intensive Care Medicine, Royal United Hospital NHS Trust, Bath, UK; d Bristol Biomedical Research Centre, University of Bristol NHS Foundation Trust and University of Bristol, Bristol, UK; e School of Chemistry, University of Bristol, Bristol, UK

**Keywords:** Aerosol, Suction, Upper airway, Open suctioning

## Abstract

**Background:**

Open respiratory suctioning is defined as an aerosol generating procedure (AGP). Laryngopharyngeal suctioning, used to clear secretions during anaesthesia, is widely managed as an AGP. However, it is uncertain whether upper airway suctioning should be designated as an AGP due to the lack of both aerosol and epidemiological evidence.

**Aim:**

To assess the relative risk of aerosol generation by upper airway suctioning during tracheal intubation and extubation in anaesthetized patients.

**Methods:**

This prospective environmental monitoring study was undertaken in an ultraclean operating theatre setting to assay aerosol concentrations during intubation and extubation sequences, including upper airway suctioning, for patients undergoing surgery (*N*=19). An optical particle sizer (particle size 0.3–10 μm) sampled aerosol 20 cm above the patient's mouth. Baseline recordings (background, tidal breathing and volitional coughs) were followed by intravenous induction of anaesthesia with neuromuscular blockade. Four periods of laryngopharyngeal suctioning were performed with a Yankauer sucker: pre-laryngoscopy, post-intubation, pre-extubation and post-extubation.

**Findings:**

Aerosol was reliably detected {median 65 [interquartile range (IQR) 39–259] particles/L} above background [median 4.8 (IQR 1–7) particles/L, *P*<0.0001] when sampling in close proximity to the patient's mouth during tidal breathing. Upper airway suctioning was associated with a much lower average aerosol concentration than breathing [median 6.0 (IQR 0–12) particles/L, *P*=0.0007], and was indistinguishable from background (*P*>0.99). Peak aerosol concentrations recorded during suctioning [median 45 (IQR 30–75) particles/L] were much lower than during volitional coughs [median 1520 (IQR 600–4363) particles/L, *P*<0.0001] and tidal breathing [median 540 (IQR 300–1826) particles/L, *P*<0.0001].

**Conclusion:**

Upper airway suctioning during airway management was not associated with a higher aerosol concentration compared with background, and was associated with a much lower aerosol concentration compared with breathing and coughing. Upper airway suctioning should not be designated as a high-risk AGP.

## Introduction

Airway suctioning is a potentially life-saving medical intervention performed in a wide range of settings both within hospitals and in the community. ‘Respiratory tract suctioning’ and ‘open suctioning of airways’ are classified as aerosol generating procedures (AGPs) based on weak epidemiological data suggesting increased risk of transmission during the outbreak of severe acute respiratory syndrome in 2003 [[1], [2], [3]]. The UK AGP guidelines state ‘the consensus view of the UK IPC [infection prevention and control] cell is that only open suctioning beyond the oropharynx is currently considered an AGP’ [3]. A recent systematic review concluded it was ‘not possible to establish a clear absence of risk associated with [airway suctioning]’ due to the lack of evidence [4]. Specifically, there are no clinical aerosol studies to help inform any risk assessments for airway suctioning.

**Figure 1:**
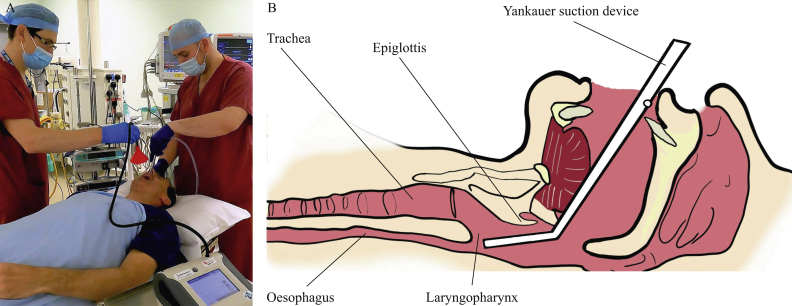
(A) Mock-up of aerosol sampling approach during upper airway suctioning. The funnel was held 20 cm away from the patient's face during baseline measures and from pre-oxygenation until completion of post-intubation suctioning. The funnel was also positioned 20 cm away from the mouth before tracheal extubation to sample during pre- and post-extubation suctioning. (B) Sagittal cut-through of the upper airway to demonstrate the position of the Yankauer suction tip in the laryngopharynx during upper airway suctioning.

The clearance of airway secretions is an essential part of both elective and emergency airway management. Placement of a Yankaeur-type sucker into the laryngopharynx to clear secretions around the glottic inlet prior to tracheal intubation and following extubation constitutes open suctioning beyond the oropharynx (Figure 1). As such, this intervention, which is commonly performed during tracheal intubation/extubation sequences, is managed as an AGP, which necessitates the use of airborne personal protective equipment (PPE) and fallow time after its performance. With over 1.2 million general anaesthetics performed using a tracheal tube per year in the UK alone, upper airway suctioning during general anaesthesia provides a useful model to determine the risk of aerosol generation associated with this medical procedure [5].

Clinical aerosol monitoring was undertaken during tracheal intubation and extubation sequences with pre-specified periods of upper airway suctioning in patients undergoing general anaesthesia for surgery. Aerosol generated during upper airway suctioning was compared with the patient's own coughs and breathing to assess the relative risk associated with the procedure.

## Methods

This study was performed as part of the National Institute for Health Research (NIHR)-funded MAGPIE study (within the AERATOR group of studies). Ethical approval was granted by Greater Manchester Research Ethics Committee (Ref. 20/NW/0393, approved 18/09/2020) and all patients gave written informed consent. The AERATOR study was granted Urgent Public Health status by NIHR and is registered in the ISRCTN registry (ISRCT N21447815).

A prospective environmental monitoring study was undertaken in a UK hospital (Southmead Hospital, North Bristol NHS Trust). All recordings were undertaken in operating theatres with ultraclean ventilation systems (EXFLOW 32, Howorth Air Technology, Farnworth, UK) placed in standby mode. A detailed description of the effect of placing this ventilation system in standby mode has been published previously [6,7]. This results in an ultraclean environment with an air exchange rate equivalent to standard UK operating theatres.

Aerosol monitoring was performed using an optical particle sizer (OPS) (Model 3330; TSI Inc., Shoreview, MN, USA) which detects particles with diameter from 0.3 to 10 μm, reporting the recorded size distribution, number and mass concentrations over a 1-s interval. A three-dimensional printed funnel [90-mm height, maximum diameter 75 mm, 10-mm exit port, formed of polylactic acid on a RAISE3D Pro2 Printer (3DGBIRE, Chorley, UK)] was attached to the OPS via a 1.25-m length of conductive silicone tubing (TSI Inc.). The authors have used this sampling methodology in previous studies investigating intubation, facemask ventilation, supraglottic airway use and oesophagoduodenoscopy [6,[8], [9], [10]]. The performance characteristics and aerosol sampling losses in the funnel/tubing have been characterized previously and shown to be relatively minor for respirable particles [11].

All patients were >18 years of age and were scheduled for either elective (*N*=11) or emergency (*N*=8) surgery. The elective patients were on low-risk ‘green’ pathways with respect to coronavirus disease 2019 (COVID-19) [i.e. asymptomatic, negative COVID-19 polymerase chain reaction (PCR) test in the previous 72 h and had self-isolated since this test]. Patients undergoing emergency surgery, who were asymptomatic for COVID-19 but had not self-isolated prior to admission and either had a negative COVID-19 PCR test result or had a PCR test pending were treated as having indeterminate risk for COVID-19 (‘amber’ pathway).

All participants were positioned supine with head positioning and airway management as per the anaesthetist's preference. The research team were not involved in the provision of anaesthetic care. All members of the research and anaesthetic team wore PPE according to hospital protocol depending on the COVID-19 risk status of the patient.

Aerosol measurements of particle concentration were performed with the sampling funnel positioned 20 cm above the patient's mouth (Figure 1A). A standardized protocol was followed for each patient.

Baseline aerosol sampling (performed before induction of anaesthesia) comprised measuring background (with the funnel directed away from the patient), 60 s of tidal breathing and three volitional coughs.

After pre-oxygenation with 100% oxygen, anaesthesia was induced with intravenous propofol plus an opioid, followed by neuromuscular blockade. Four phases of upper airway suctioning were specified:

• pre-intubation: immediately before laryngoscopy (after neuromuscular blockade);
• post-intubation: immediately after tracheal intubation (after cuff inflation);
• pre-extubation: before tracheal extubation (before cuff deflation); and
• post-extubation: after tracheal extubation (if tolerated by the patient).

Upper airway suctioning was performed using a Yankauer suction device (with the side-port occluded), inserted via the mouth to the level of the laryngopharynx with the tip positioned above the vocal cords (Figure 1B). All periods of upper airway suctioning were performed for a minimum of 10 s. The Yankauer sucker was connected to an active negative pressure suction cannister as part of the anaesthetic machine (Aisys CS2, GE Healthcare, Chicago, IL, USA) set to maximum (-35 kPa). Additional suctioning was undertaken as clinically indicated.

**Figure 2:**
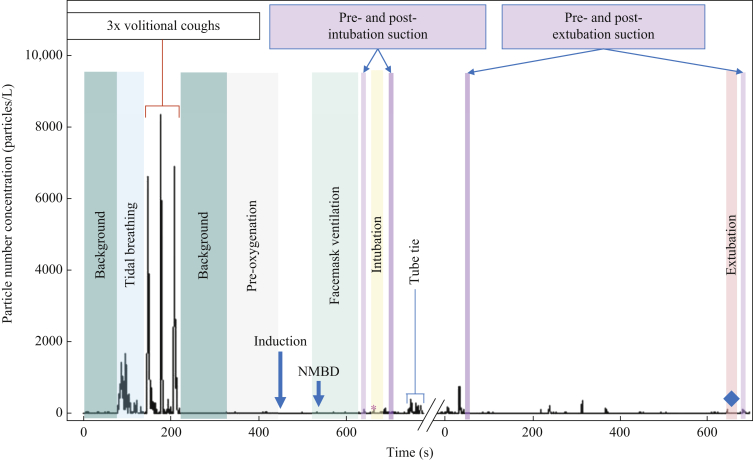
Timeline of aerosol concentration associated with upper airway suctioning during the intubation and extubation sequences in a representative patient. This shows the concentration of particles detected during baseline respiratory manoeuvres (tidal breathing and voluntary coughs), background monitoring, facemask ventilation, intubation (from laryngoscopy until inflation of tracheal tube cuff), extubation (tracheal tube cuff down until tube out) and phases of suctioning. Note break in recording shown on x-axis between intubation (indicated by asterisk) and extubation (indicated by diamond) sequences. NMBD, neuromuscular blocking drug. Particle size range 0.3–10 μm.

Sampling was continuous during the whole intubation sequence from baseline breathing measures until the second period of upper airway suctioning following tracheal intubation (Figure 2). Aerosol sampling was recommenced prior to tracheal extubation to include both periods of upper airway suctioning.

All events were timestamped by the researcher. These included: baseline tidal breathing; coughs; induction of anaesthesia; neuromuscular blockade administration; facemask ventilation; airway adjunct insertion; each period of airway suctioning; laryngoscopy; tracheal intubation; tracheal cuff inflation; tracheal cuff deflation; and tracheal extubation.

The clinically significant aerosol emission rate with regard to risk of respiratory pathogen transmission, and how this is reflected in the concentration of aerosol at the source, are currently unknown. In the authors' facemask ventilation study, the mean [standard deviation (SD)] particle concentration for tidal breathing was 303 (SD 375) particles/L. A two-fold increase in the average aerosol concentration above breathing was considered to be potentially meaningful (albeit still considerably lower than that associated with a cough). A-priori sample size calculations for a non-parametric paired comparison predicted that recordings of complete data on 12 participants would ensure that the study was adequately powered to detect a difference of this magnitude when compared with the patient's own tidal breathing (80% power, alpha 0.05, G∗Power3.1.9.4).

TSI Aerosol Instrument Manager was used to process the aerosol data prior to analysis in Origin Pro (Originlab, Northampton, MA, USA) and R Version 4.1.2. The Shapiro–Wilk test was used to assess the normality of data. Comparisons were made between aerosol measurements using a repeated measures analysis for non-parametric data (Friedman with Dunn's multiple comparisons with Bonferroni correction). Separate analyses were performed for the intubation sequences (*N*=18), extubation sequences (*N*=13) and peak values (*N*=19) to account for missing values. The significance level was set at *P*<0.05. All data are presented as mean (SD) or median [interquartile range (IQR)].

## Results

This aerosol monitoring study was performed over a 7-week period during anaesthesia for patients undergoing orthopaedic, neurosurgical and general surgical procedures. Following informed consent, 19 patients were recruited [nine females, mean age 70 (SD 10) years, mean body mass index 29.1 (SD 5.9) kg/m^2^].

The airway of each patient was managed with a cuffed oral tracheal tube. During facemask ventilation (before tracheal intubation), four patients had a Guedel oropharyngeal airway inserted to facilitate ventilation. Neuromuscular blockade was provided with rocuronium (*N*=18) or atracurium (*N*=1).

All patients (*N*=19) underwent pre-intubation and post-intubation upper airway suctioning. Fourteen patients underwent pre-extubation upper airway suctioning, and all but one of these patients tolerated post-extubation upper airway suctioning. Therefore, during the conduct of the study, aerosol generation was conducted through 19 entire intubation sequences (including periods of facemask ventilation) and 14 extubation sequences. In total, 784 s of aerosol data were recorded during episodes of suctioning (410 s peri-intubation, 374 s peri-extubation).

In eight instances, tracheal tube placement required the use of an adjunct: a bougie in seven cases and a stylet in one case. One patient had a rapid sequence induction of anaesthesia for emergency surgery and no period of facemask ventilation, but did have pre-intubation suctioning. In one patient, an accidental oesophageal intubation was identified following cuff inflation and absence of CO_2_ noted on capnography. This patient had the tracheal tube removed, underwent a further period of facemask ventilation, and was intubated successfully at the second attempt with the assistance of a bougie but without any further suctioning. Both intubation attempts have been included in the analysis, and post-intubation suctioning was performed after the airway had been secured. One patient coughed twice following tracheal extubation: once immediately after extubation and once during post-extubation suctioning.

**Figure 3:**
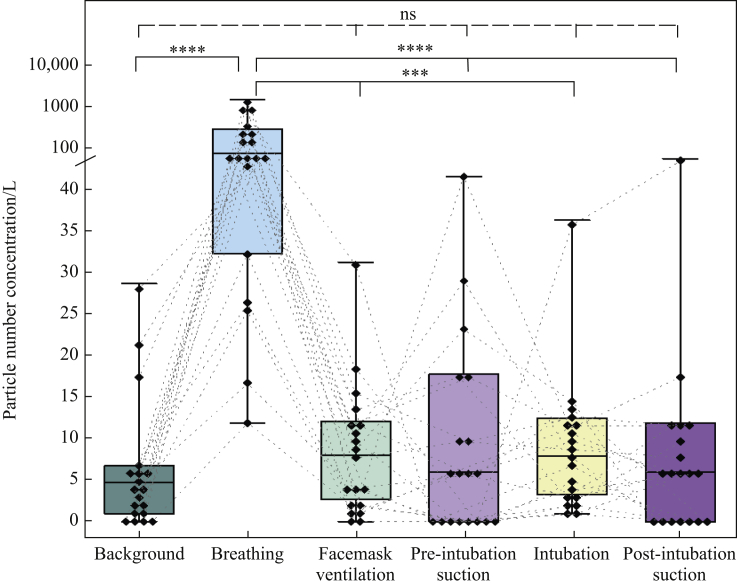
Plot of aerosol concentrations for the phases of the tracheal intubation sequence including suctioning compared with breathing and background aerosol concentrations. Both episodes of airway suctioning were associated with a lower aerosol concentration than tidal breathing (likewise for facemask ventilation and intubation). Boxes represent interquartile ranges, whiskers represent ranges, solid horizontal line represents median, diamonds represent individual patient data, and dotted lines connect individual patient's data points [*N*=19 except facemask ventilation (*N*=18); ∗∗∗∗*P*<0.0001; ∗∗∗*P*<0.001; ns, non-significant; Friedman with Dunn's multiple comparisons]. Note linear-log scale representing aerosol concentrations to cover the wide range of values seen with tidal breathing.

**Figure 4:**
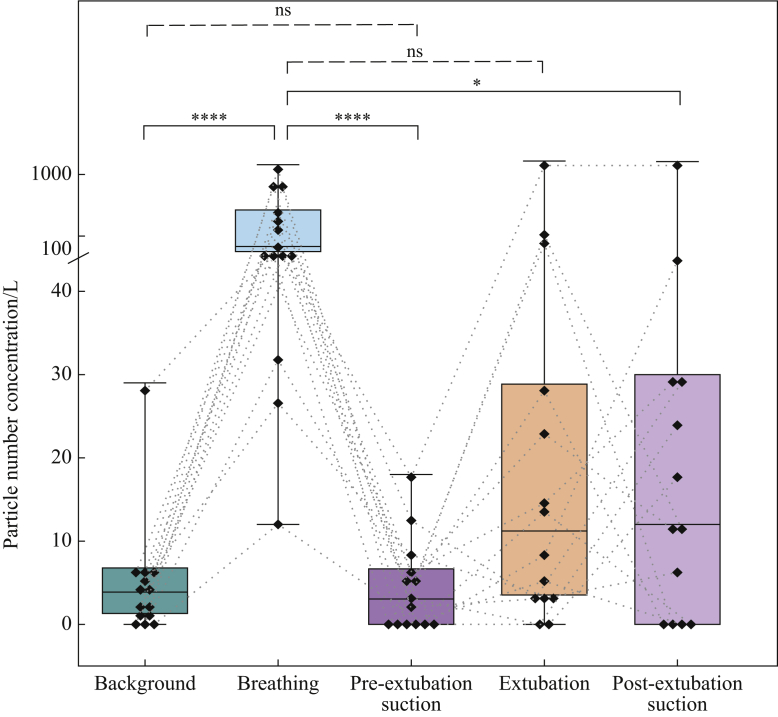
Aerosol concentrations for the phases of tracheal extubation including suctioning compared with tidal breathing and background. Upper airway suctioning with a secured (intubated) airway was associated with a lower aerosol concentration than tidal breathing, and was no different to background. Upper airway suctioning in an open airway in a spontaneously breathing patient was not significantly different to that seen with tidal breathing. Boxes represent interquartile ranges, whiskers represent ranges, solid horizontal line represents median, diamonds represent individual patient's data, dotted lines connect individual patient's data points [*N*=14 except post-extubation suctioning (*N*=13); ∗∗∗∗*P*<0.0001; ∗*P*<0.05; ns, non-significant; Friedman with Dunn's multiple comparisons]. Note linear-log scale representing aerosol concentrations to cover the wide range of values seen with tidal breathing.

Median background aerosol concentration was 4.8 (IQR 1.0–6.8) particles/L. Aerosol generated by tidal breathing was reliably detected above background [median 65 (IQR 39–259] particles/L), *P*<0.0001, Friedman] (Figure 2, Figure 3, Figure 4). Aerosol concentrations detected during periods of facemask ventilation, pre-intubation suctioning, tracheal intubation (from laryngoscopy until cuff-up) and post-intubation suctioning were not significantly different from background (all *P*>0.99) or each other (Table I and Figure 3). Aerosol concentrations detected during suctioning pre- and post-tracheal intubation and pre-tracheal extubation were far lower than aerosol concentrations detected during tidal breathing (all *P*<0.0001, Friedman) (see Table I and Figure 3, Figure 4).

**Table I:** Aerosol concentrations measured during different procedures compared with tidal breathing and background (Friedman with Dunn's multiple comparisons) [median (interquartile range]

Event	*N*	Aerosol concentration (particles/L)	vs breathing (*P*-values)	vs background (*P*-values)
Background	19	4.8 (1.1–6.8)	<0.0001	-
Tidal breathing	19	65 (39–259)	-	<0.0001
Facemask ventilation	18	8.5 (2.8–12)	0.0002	>0.99
Pre-intubation suctioning	19	6.0 (0–14)	<0.0001	>0.99
Tracheal intubation	19	8.0 (3.5–12)	0.0002	>0.99
Post-intubation suctioning	19	6.0 (0–11)	<0.0001	>0.99
Pre-extubation suctioning	14	3.1 (0–6.5)	<0.0001	>0.99
Extubation	14	11 (3.6–28)	0.1	0.6
Post-extubation suctioning	13	12 (0–30)	0.029	>0.99

Aerosol concentrations recorded during extubation [median 11 (IQR 3.5–12) particles/L] and post-extubation suctioning [median 12 (IQR 0–30) particles/L] showed a wide spread of values (Figure 4); however, extubation aerosol concentrations were not significantly different to awake tidal breathing, and post-extubation suctioning was lower than tidal breathing (*P*=0.1 and P = 0.029, respectively, Friedman). The median aerosol concentration recorded during all periods of suctioning was 6.0 (IQR 5–30) particles/L, which was much lower than that for tidal breathing (*P*=0.0007, Friedman).

**Figure 5:**
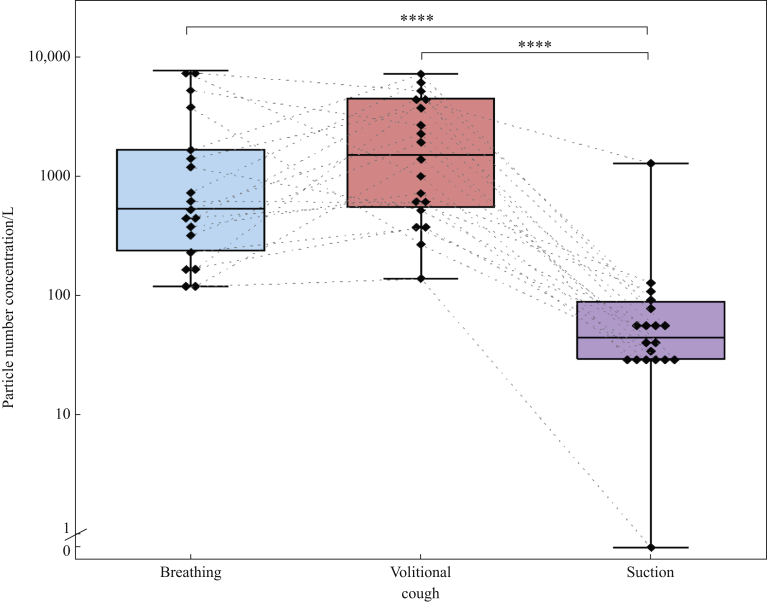
Peak aerosol concentrations during breathing and coughing compared with peak aerosol concentration measured during all periods of airway suctioning. Boxes represent interquartile ranges, whiskers represent ranges, dotted lines connect individual patient's data points, solid horizontal line represents median, diamonds represent individual patient's data (*N*=19; ∗∗∗∗*P*<0.0001; Friedman with Dunn's multiple comparisons).

The peak aerosol concentrations produced by volitional coughs [median 1520 (IQR 600–4363) particles/L] and tidal breathing [median 540 (IQR 240–1680) particles/L] were many-fold higher than the peak concentrations recorded during all periods of upper airway suctioning [median 45 (IQR 30–90) particles/L, both *P*<0.0001, Friedman) (Figure 5).

## Discussion

Managing upper airway suctioning as an AGP impacts theatre efficiency; when performed before tracheal extubation, all healthcare workers present in the operating theatre must either wear airborne protection PPE or leave the operating theatre. Evacuation of team members at the end of the case prevents theatre cleaning and preparation for the next patient, until a period of ‘fallow time’ has been observed. This study shows that upper airway suctioning in an anaesthetized patient is associated with aerosol concentrations that are indistinguishable from background, and much lower than those seen during tidal breathing and coughing. Upper airway suctioning in an anaesthetized patient should therefore not be designated or managed as an AGP. When upper airway suctioning is performed following tracheal extubation, when a patient is breathing and able to cough, the aerosol concentrations generated are no higher than those produced by natural respiratory events in the same patient. Upper airway suctioning in the awake patient does not, in itself, generate aerosol, and adds no additional risk to the patient's own natural respiratory activities.

The application of upper airway suctioning of the laryngopharynx in anaesthetized patients with a secured airway (tracheal tube *in situ*) represents the best approximation of studying the procedure in isolation, independent of any aerosol generation from the patient's own respiratory activities. This is represented in this study by airway suctioning post-intubation and pre-extubation. It is reassuring that no increase in aerosol concentration was observed during these suction procedures, indicating that upper airway suctioning is not intrinsically an AGP; this finding is generalizable to a wide range of clinical settings outside anaesthetic airway management.

This study found that upper airway suctioning of the open airway in a spontaneously breathing patient (after extubation) does not increase the level of aerosol generated above that produced by the patient's own natural respiratory activities. In the current study, one patient coughed following tracheal extubation and again during the subsequent period of suctioning, which generated aerosol. Patients may cough during or following any airway manipulation. If this natural respiratory activity is of concern to the practitioner (due to a risk assessment indicating potential risk of COVID-19), it is suggested that all close patient interactions should be performed whilst wearing airborne protection PPE as the patient will be generating respiratory aerosol with every breath they exhale [7,12,13]. It should also be noted that placement of a facemask over a patient's mouth and nose will effectively mitigate against the escape of aerosol generated during a cough after extubation.

This study extends the clinical aerosol methodology developed by the AERATOR group. The strengths of this study include the use of continuous aerosol sampling during airway management with event timestamping, which enables accurate comparison of the components of airway management for each patient; within-patient comparisons account for the known large variation between subjects; and the ultraclean theatre environment enables resolution of respiratory aerosols from breathing and coughing above background. These results reflect real-world practice having captured airway management requiring oropharyngeal airway devices, intubation aids and management of an unintended oesophageal intubation. Additionally, one patient had hiccoughs following tracheal intubation, and bit down on the tracheal tube during extubation for >90 s. There was no increase in aerosol concentration during any of these events. The procedures were performed by anaesthetists ranging from novice to consultant; as such, the findings are generalizable to a wide variety of settings.

The study has some weaknesses. It was a single-centre study, and the number of patients was relatively small; however, within-subject comparison adequately powered this study to be able to detect whether aerosol concentrations during suctioning were increased above the level generated during tidal breathing. Aerosol measurements were conducted during routine care of a convenience sample of patients; nonetheless, their age distribution and body mass indexes were representative of the surgical population. There are challenges inherent in distinguishing respiratory aerosols from other sources of airborne particles, but the ultra-low background aerosol concentration and the high-temporal resolution sampling enabled clear identification of events. Additionally, the distinctive particle size distribution led to confidence that the majority of recorded aerosol was from the respiratory tract. The authors have previously identified artefactual sources of aerosol, and sought to minimize their occurrence (e.g. particles generated from manipulation of pillows or gauze) [10]. The authors were unable to identify whether the recorded aerosol was carrying virus, or make an absolute assessment regarding the risk of transmission. Of note, the degree of risk associated with absolute viral levels is currently unknown.

There seems to be no plausible mechanism for aerosol generation by upper airway suctioning. Suction canisters operate by applying a sub-atmospheric pressure, and are designed to draw secretions into the tubing and away from the site of the suction device, and therefore away from the practitioner. The use of suctioning in the oropharynx has been shown to reduce the amount of aerosol generated during other AGPs such as oesophagoduodenoscopy and dental procedures [[15], [16], [17], [18]]. In contrast, upper airway suctioning performed in a non-anaesthetized patient may evoke coughing, which can generate high levels of aerosol. As such, the procedure is not aerosol generating, but the patient can generate respiratory aerosol through natural respiratory activities. These observations can provide some reassurance to healthcare practitioners working in other settings, such as intensive care, resuscitation areas, endoscopy suites and geriatric wards, where oro-/laryngopharyngeal suctioning is commonly required, and the findings indicate that such suctioning adds no additional aerosol risk above that resulting from the patient's own respiratory activities (i.e. breathing, talking and coughing) which should be subject to a patient-focused risk assessment regarding transmission risks for airborne pathogens (see below).

During the conduct of this study, aerosol generation was recorded through 19 entire intubation sequences and 14 extubation sequences (including periods of facemask ventilation), which produced findings consistent with the authors' previous study with low levels of aerosol generation compared with cough [8,9]. In total, 19 patients were recruited to ensure that the study was adequately powered to detect differences during post-extubation suctioning, which was more difficult to obtain (*N*=13). The current study extends the authors' previous findings by applying more stringent criteria through sampling aerosol from closer to the patient (20 cm vs 50 cm), and by comparing the patient's own tidal breathing and coughs against procedural aerosol generation. In all cases, this confirmed that tracheal intubation, extubation and facemask ventilation are low risk for aerosol generation. Thus, in combination with the observation that upper airway suctioning is not aerosol generating, the authors' confidence has increased to assert that none of the common components of tracheal intubation and extubation sequences in anaesthetized patients should be considered as AGPs.

These findings add further weight to the need to reassess the list of AGPs, and may be used to inform UK infection prevention and control guidance [3,13]. This places the emphasis of risk on the medical procedure (many of which have been demonstrated not to generate aerosol [9,[19], [20], [21], [22]]) whilst neglecting multiple factors that should be considered during the risk of assessment of any patient interaction. The risk assessment should consider the likelihood of the patient being infected with a respiratory pathogen (such as severe acute respiratory syndrome coronavirus-2); the proximity of healthcare workers to the patient's respiratory tract; the duration of this proximity; the health, immune and vaccination status of the healthcare workers present; and the environment in which the interaction occurs. The results of this study lead to the conclusion that the focus and emphasis regarding the transmission risk of aerosolized pathogens should be on the patient rather than specifically on the procedure.

In conclusion, upper airway suctioning during airway management is not associated with a higher aerosol concentration than background, and was associated with a much lower aerosol concentration compared with breathing and coughing. Upper airway suctioning should not be designated as a high-risk AGP.
